# First trimester PAPP-A levels correlate with sFlt-1 levels longitudinally in pregnant women with and without preeclampsia

**DOI:** 10.1186/1471-2393-13-85

**Published:** 2013-04-04

**Authors:** Aditi R Saxena, Ellen W Seely, Janet W Rich-Edwards, Louise E Wilkins-Haug, S Ananth Karumanchi, Thomas F McElrath

**Affiliations:** 1 Department of Medicine, Brigham and Women’s Hospital, 221 Longwood Avenue, RFB-2, Boston, MA, 02115, USA; 2 Department of Obstetrics and Gynecology, Brigham and Women’s Hospital, Boston, MA, USA; 3 Department of Medicine, Beth Israel Deaconess Medical Center, Boston, MA, USA

## Abstract

**Background:**

First trimester Pregnancy Associated Plasma Protein A (PAPP-A) levels, routinely measured for aneuploidy screening, may predict development of preeclampsia. This study tests the hypothesis that first trimester PAPP-A levels correlate with soluble fms-like tyrosine kinase-1 (sFlt-1) levels, an angiogenic marker associated with preeclampsia, throughout pregnancy.

**Methods:**

sFlt-1 levels were measured longitudinally in 427 women with singleton pregnancies in all three trimesters. First trimester PAPP-A and PAPP-A Multiples of Median (MOM) were measured. Student’s T and Wilcoxon tests compared preeclamptic and normal pregnancies. A linear mixed model assessed the relationship between log PAPP-A and serial log sFlt-1 levels.

**Results:**

PAPP-A and PAPP-A MOM levels were significantly lower in preeclamptic (n = 19), versus normal pregnancies (p = 0.02). Although mean third trimester sFlt-1 levels were significantly higher in preeclampsia (p = 0.002), first trimester sFlt-1 levels were lower in women who developed preeclampsia, compared with normal pregnancies (p = 0.03). PAPP-A levels correlated significantly with serial sFlt-1 levels. Importantly, low first trimester PAPP-A MOM predicted decreased odds of normal pregnancy (OR 0.2, p = 0.002).

**Conclusions:**

Low first trimester PAPP-A levels suggests increased future risk of preeclampsia and correlate with serial sFlt-1 levels throughout pregnancy. Furthermore, low first trimester PAPP-A status significantly predicted decreased odds of normal pregnancy.

## Background

Preeclampsia affects approximately 5% of all pregnancies and remains a significant cause of maternal and neonatal morbidity and mortality [1]. Early risk stratification may improve the identification of women at highest risk for preeclampsia and provide an opportunity for intervention [2]. Thus far, several markers have been evaluated regarding their ability to predict preeclampsia in the first trimester, prior to clinical onset of clinical signs. However, the accuracy of these markers are suboptimal for clinical use [3,4]. Pregnancy associated Plasma Protein A (PAPP-A) levels are measured in clinical practice for the 1^st^ trimester aneuploidy screen, as low levels indicate increased risk for trisomy 13, 18, 21 [5]. Emerging data support that preeclampsia and other pregnancy complications are also associated with low PAPP-A levels [6,7]. Soluble fms-like tyrosine kinase 1 (sFlt-1) is a circulating antagonist to vascular endothelial growth factor (VEGF) and an anti-angiogenic factor in placentation. Although not routinely applied in clinical settings, sFlt-1 levels are markedly increased in the second and third trimesters in cases of preeclampsia. Furthermore, the rise in sFlt-1 precedes the onset of clinical signs of preeclampsia [8]. As both PAPP-A and sFlt-1 may predict increased risk for preeclampsia, in this study we explore the relationship of 1^st^ trimester PAPP-A levels, which are obtained routinely for clinical care, with serial levels of sFlt-1, a widely accepted marker of preeclampsia in research, and test the hypothesis that 1^st^ trimester PAPP-A correlates with serial sFlt-1 levels throughout pregnancy. In addition, the relationship of first trimester PAPP-A to serial levels of placental growth factor (PlGF) and serial ratios of sFlt-1 to PlGF were also explored, as studies have shown that PlGF is significantly lower in preeclampsia [9] and the ratio of sFlt-1/PlGF is also significantly elevated in preeclampsia [10,11], prior to the onset of clinical signs.

## Methods

### Study population

In this nested case-control study, study participants (n = 427) were chosen from a prospectively collected, longitudinal cohort of pregnant women, initiating prenatal care between October 2006 and September 2008 at Brigham and Women’s Hospital in Boston, MA. This hospital served as one of three study sites for a larger study to explore the utility of angiogenic markers in early diagnosis of preeclampsia [4]. Blood was drawn from subjects at four clinical visits during pregnancy: at 8–10 weeks estimated gestational age (EGA), 17–19 weeks EGA, 23–25 weeks EGA, and 34–35 weeks EGA. Pregnancy related diagnoses were abstracted from medical records. A panel of expert obstetricians confirmed the diagnosis of preeclampsia by review of medical records, using Working Group criteria [12]. These experts were blinded to the angiogenic factor and PAPP-A measurements for each subject. Subjects were eligible for inclusion in the study if they were a minimum of 18 years of age and pregnant with an estimated gestational age less than or equal to 15 weeks. This study received approval from the Institutional Review Board at Brigham and Women’s Hospital. Subjects signed consent prior to initiation of study directed activities.

For this analysis, participants were included if they had completed a 1^st^ trimester screening with PAPP-A and human chorionic gonadotropin (hCG) levels and were later diagnosed with either preeclampsia or normal pregnancy. Women with other pregnancy complications, such as gestational hypertension, gestational diabetes, or preterm delivery without the diagnosis of preeclampsia, were not included in this analysis. Subjects were excluded if they had any medical diagnoses prior to pregnancy, including diabetes or hypertension prior to pregnancy, with the exception of use of thyroid medication. Subjects were also excluded if they required *in vitro* fertilization to achieve pregnancy. Race was self-defined. Smoking status was self-reported.

### Study methods

In each woman, body mass index (BMI), mean arterial pressure (MAP), plasma sFlt-1 levels, and plasma levels of placental growth factor (PlGF) were measured at four clinical visits during pregnancy, at time points described earlier. PAPP-A and hCG levels were obtained in each woman as part of their 1^st^ trimester screening. There were no cases of aneuploidy in this cohort. Gestational age was confirmed by ultrasound prior to 15 weeks gestation. Gestational age at delivery and birth weight were abstracted from medical records.

### Laboratory assays

Specimens were processed within 4 hours of venipuncture in a refrigerated centrifuge and stored at −80°C until analysis. sFlt-1 and PlGF were measured using prototype ARCHITECT immunoassays (Abbott Laboratories, Abbott Park, IL), which are chemiluminescent microparticle immunoassays. The sFlt-1 immunoassay has a lower limit of detection of 0.10 ng/mL, ranging to 150 ng/mL. The intra- and interassay coefficients of variation (CV) were less than 7%. The PlGF immunoassay has a lower limit of detection of 1 pg/mL with a range up to 1,500 pg/mL. The combined intra- and interassay coefficients of variation (CV) were less than 7%.

PAPP-A and total hCG levels were obtained from the patients’ clinical records. Tests were performed at Women and Infants Hospital, Providence, RI. PAPP-A levels were measured by ELISA (Diagnostic Systems Laboratories/Beckman Coulter, Webster, TX), with reported sensitivity of 0.21μIU/mL and CV 3.6% (Palomaki 2007). Total hCG levels were measured on the Immulite (Siemens, Los Angeles CA) with reported sensitivity of 0.23 mIU/mL and CV 5% (Palomaki 2007). Multiples of median (MOM) were reported and adjusted for gestational age, maternal weight and race.

### Statistical analysis

Age, parity, 1^st^ trimester BMI, race, and MAP in each trimester were demographic variables of interest. Age, parity, BMI, and MAP were continuous variables, whereas race was treated as a categorical variable. The Shapiro-Wilk Test was used to test for normal distribution. The Student’s *T* test was used for two-group comparisons between women with normotensive pregnancy and women with preeclampsia. For non-normally distributed data, the Wilcoxon Rank Sum test was used. Data are expressed as mean ± standard deviation (SD) or median (interquartile range) for non-normally distributed data. Non-normally distributed data were log-transformed for correlation. For calculation of sFlt-1/PlGF ratio, sFlt-1 levels were first converted to pg/mL and then expressed as a ratio to measured PlGF levels [13]. The z-score of the birthweight was calculated as ([infant birthweight-mean of birthweight at same weeks EGA]/standard deviation of mean) [14]. Unadjusted correlation of log PAPP-A MOM to serial levels of log sFlt-1 was performed individually by calculation of Pearson’s correlation coefficient. To determine effect of PAPP-A MOM on log sFlt-1, PAPP-A MOM levels were dichotomized (≥ or < the median PAPP-A MOM). Logistic regression was used to determine the ability of dichotomized PAPP-A MOM to predict normal pregnancy, adjusted for race and first trimester BMI. Odds ratios (OR) are expressed as OR, [95% confidence interval]. A p-value of less than 0.05 was significant. Analyses were performed using JMP version 9.0.0 (SAS Institute, Inc. Cary, NC).

## Results

### Subject demographics

The total number of subjects selected for this analysis was 427. Nineteen women developed preeclampsia, and 408 women were classified with normal, uncomplicated pregnancy (Table 1). There were no significant differences between women with preeclampsia and women with normal pregnancy with respect to age, parity, or self-reported smoking. EGA was lower in women with preeclampsia, but this difference did not reach statistical significance. The two groups did differ significantly in racial distribution, first trimester BMI, and MAP obtained during each trimester (Table 1). Women who developed preeclampsia had a lower prevalence of Caucasian race and higher prevalence of African-American race. These women also demonstrated higher first trimester BMI and higher MAP in all trimesters of pregnancy.

**Table 1:** Demographic characteristics of study population

**Characteristics**	**Preeclamptic pregnancies (n = 19)**	**Normal pregnancies (n = 408)**	**p-value**
Age, years	32 ± 7	31 ± 6	0.7
Parity	0.8 ± 0.9	0.9 ± 1.0	0.9
Race			
Caucasian	9 (47%)	243 (60%)	*x*^2^ = 10.3,p = 0.04
African-American	7 (37%)	51 (13%)	
Asian	0	20 (5%)	
Hispanic	3 (16%)	75 (18%)	
Unknown/Other	0	19 (5%)	
Any smoking during pregnancy	3 (16%)	27 (7%)	0.3
First trimester BMI, kg/m^2^	29.5 ± 6.4	25.2 ± 5.1	p = 0.001
First trimester MAP, mmHg	84 ± 9	79 ± 7	p = 0.02
Second trimester MAP, mmHg	83 ± 11	77 ± 8	p = 0.006
Third trimester MAP, mmHg	85 ± 24	80 ± 8	p = 0.0008
EGA at delivery, weeks	38.5 ± 2.1	39.4 ± 1.2	p = 0.08

Data presented as means ± SD, or N (%).

BMI body mass index; MAP, mean arterial pressure; EGA, estimated gestational age.

### First trimester markers and subsequent pregnancy outcome

Both measured PAPP-A levels and PAPP-A MOM levels were significantly lower in the first trimester in women who later developed preeclampsia, compared with women with normal pregnancy (Table 2). Measured hCG levels and hCG MOM levels did not differ significantly between the two groups.

**Table 2:** First trimester markers and pregnancy outcome

**Marker**	**Preeclamptic pregnancies (n = 19)**	**Normal pregnancies (n = 408)**	**p-value**
PaPP-A, mIU/mL	2.3 (1.33–3.31)	2.8 (1.9–4.2)	0.04
PAPP-A MOM	0.9 (0.7–1.0)	1.1 (0.8–1.4)	0.02
HCG, IU/mL	70.6 (50.0–95.2)	73.4 (55.7–97.6)	0.5
HCG MOM	1.0 (0.7–1.6)	1.0 (0.8–1.3)	0.7

Data presented as median (interquartile range).

MOM, multiple of median.

### Association of dichotomized PAPP-A MOM with pregnancy outcome

To explore further the significance of low 1^st^ trimester PAPP-A levels, PAPP-A MOM levels were dichotomized as low (< median) or high (≥ median). A majority of women who developed preeclampsia (n = 15 or 83%) were categorized as low PAPP-A. In contrast, women with normal pregnancy were more evenly distributed between the two groups, with 48% classified as low PAPP-A (n = 195) and 52% characterized as high PAPP-A (n = 213).

Using logistic regression adjusted for first trimester BMI, women who had lower than median PAPP-A MOM (LowPAPPAMOM) had decreased odds of having a normal pregnancy, OR 0.2, [0.04, 0.6], p = 0.002. Conversely, women with ≥ median PAPP-A (HighPAPPAMOM), had significantly increased odds of having a normal pregnancy, OR 5.5, [1.8, 24.3], p = 0.002.

### Serum angiogenic markers and subsequent pregnancy outcome

Serum sFlt-1 levels obtained in the 1^st^ trimester were significantly lower in women who later developed preeclampsia, compared with women with normal pregnancy, and did not differ significantly at 17–19 weeks or at 23–25 weeks between the two groups (Table 3). However, at 34–35 weeks, sFlt-1 levels were significantly higher in women who developed preeclampsia. In contrast, PlGF levels appeared to be higher in the 1^st^ trimester, and lower at 17–19 weeks, 23–25 weeks, and 34–35 weeks in women who later developed preeclampsia, compared with women with normal pregnancy, however these differences did not reach statistical significance. The sFlt-1/PlGF ratio varied between the groups throughout pregnancy. In the first trimester, the sFlt-1/PlGF ratio was significantly lower in women who later developed preeclampsia, compared to women with normal pregnancy. There were no differences in sFlt-1/PlGF ratio between the two groups at 17–19 weeks or 23–25 weeks. However, at 34–35 weeks, women who developed preeclampsia had a significantly higher sFlt-1/PlGF ratio (Table 3).

**Table 3:** Serial angiogenic markers in pregnancy and pregnancy outcome

**Time point of measurement (EGA)**	**Angiogenic marker**	**Preeclamptic pregnancies (n = 19)**	**Normal pregnancies (n = 408)**	**p-value**
	sFlt-1	4.1 (2.3–5.5)	5.4 (3.5–7.5)	0.03
8–10 weeks	PlGF	24.1 (15.2–43.0)	20.7 (15.0–29.5	0.5
	sFlt-1/PlGF ratio	155.3 (85.5–195.7)	235.3 (147.2–343.5)	0.002
	sFlt-1	5.7 (3.2–9.2)	6.3 (3.9–9.0)	0.6
17–19 weeks	PlGF	112.8 (81.7–175.6)	136.4 (100.5–192.7)	0.3
	sFlt-1/PlGF ratio	35.8 (26.4–66.9)	47.1 (28.2–74.7)	0.6
	sFlt-1	6.3 (3.0–9.7)	5.7 (3.9–8.9)	0.8
23–25 weeks	PlGF	354.2 (182.6–583.1)	451.3 (303.9–651.0)	0.07
	sFlt-1/PlGF ratio	13.9 (8.5–32.2)	12.6 (7.9–21.5)	0.2
	sFlt-1	16.7 (9.0–26.7)	9.2 (6.4–13.7)	0.002
34–35 weeks	PlGF	247.9 (102.2–589.9)	372.4 (186.1–707.1)	0.09
	sFlt-1/PlGF ratio	74.0 (14.7–261.6)	25.3 (10.3–64.2)	0.03

Data presented as median (interquartile range).

sFlt-1 in ng/mL; PlGF in pg/mL; for calculation of sFlt-1/PlGF ratio, sFlt-1 was first converted to pg/mL.

### Association of dichotomized angiogenic markers with pregnancy outcome

As first trimester sFlt-1 levels exhibited similar distribution to PAPP-A MOM, these levels were also dichotomized as < median (LowSFlt-1) or ≥ median (HighSFlt-1) to determine if first trimester sFlt-1 levels could predict pregnancy outcome. However, LowSFlt-1 was not significantly associated with decreased odds of normal pregnancy, OR 0.5, [0.2, 1.4], nor was HighSFlt-1 significantly associated with increased odds of normal pregnancy, OR 2.0, [0.7, 6.6].

In contrast, dichotomized sFlt-1/PlGF ratio was associated with pregnancy outcome. Specifically, low first trimester sFlt-1/PLGF ratio (< median sFlt-1/PlGF ratio) was significantly associated with decreased odds of normal pregnancy, OR 0.23, [0.05, 0.74], p = 0.01. High first trimester sFlt-1/PLGF ratio (≥ median sFlt-1/PlGF ratio) was associated with increased odds of normal pregnancy, OR 4.29, [1.36, 18.94], p = 0.01.

### Correlation of PAPP-A with serial sFlt-1 levels

A significant positive correlation was observed between log-transformed PAPP-A MOM and log sFlt-1 in women with normal pregnancy. In these women, 1^st^ trimester PAPP-A MOM correlated positively with sFlt-1 at all four time points during pregnancy (Table 4). For women with preeclampsia, the correlation was also positive, but only approached statistical significance at 17–19 weeks. Log-transformed PAPP-A MOM was not significantly associated with log PlGF (data not shown).

**Table 4:** **Correlation of 1**^**st **^**trimester log PAPP-A MOM with log sFlt-1 at serial time points (unadjusted)**

**Time point of sFlt-1 measurement (EGA)**	**Preeclamptic pregnancies (n = 18)**^**a**^		**Normal pregnancies(n = 405)**^**b**^	
8–10 weeks	*r* = 0.35	p = 0.2	*r* = 0.26	p <0.0001
17–19 weeks	*r* = 0.50	p = 0.05	*r* = 0.32	p <0.0001
23–25 weeks	*r* = 0.33	p = 0.2	*r* = 0.27	p <0.0001
34–35 weeks	*r* = 0.18	p = 0.5	*r* = 0.19	p = 0.0002

^a^For PE pregnancies at time point 17–19 weeks, n = 16.

^b^For normal pregnancies n = 382, 390 and 394 for time points 17–19 weeks, 23–25 weeks, and 34–35 weeks, respectively.

*r*, Pearson’s correlation coefficient.

### Association of dichotomized PAPP-A MOM with serial angiogenic markers

**Figure 1:**
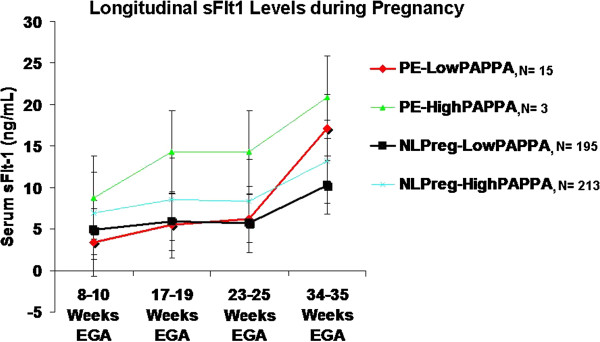
**Longitudinal sFlt-1 levels during pregnancy by PAPP-A status and pregnancy diagnosis.** PAPP-A MOM levels were dichotomized: low PAPP-A MOM (< median PAPP-A MOM) or high PAPP-A (≥ median PAPP-A MOM). A majority of women with preeclampsia were classified as low PAPP-A MOM (PE-LowPAPPA). Women with PE-LowPAPPA had the greatest change in sFlt-1 levels between the 1^st^ and 3^rd^ trimesters, compared with women with normal pregnancy, regardless of PAPP-A status (NLPreg-LowPAPPA and NLPreg-HighPAPPA, p < 0.02).

In the 1^st^ trimester, women with low PAPP-A who had a normal pregnancy (NlPreg-LowPAPPA) had similar sFlt-1 levels to women with low PAPP-A who developed preeclampsia (PE-LowPAPPA), 4.9 ± 2.7 vs. 3.4 ± 1.8 ng/mL, respectively (Figure 1). Similarly, in women with normal pregnancy who had high PAPP-A (NlPreg-HighPAPPA), 1^st^ trimester sFlt-1 did not differ significantly from women with high PAPP-A levels who later developed preeclampsia (PE-HighPAPPA), 6.9 ± 4.0 vs. 8.8 ± 3.2 ng/mL respectively. There were significant differences in 1^st^ trimester sFlt-1 between NlPreg-LowPAPPA and NlPreg-HighPAPPA, 4.9 ± 2.7 vs. 6.9 ± 4.0 ng/mL, respectively, p < 0.0001. PE-LowPAPPA also had significantly lower 1^st^ trimester sFlt-1 compared with NlPreg-HighPAPPA (3.4 ± 1.8 vs. 6.9 ± 4.0 ng/mL, p = 0.0009).

At 17–19 weeks, PE-LowPAPPA women continued to have lower sFlt-1 levels compared with PE-HighPAPPA (5.5 ± 3.3 vs. 14.3 ± 4.9 ng/mL, p = 0.02), Figure 1. In addition, NlPreg-LowPAPPA women had significantly lower sFlt-1 levels compared with NlPreg-HighPAPPA (5.9 ± 3.0 vs. 8.6 ± 5.7, p < 0.0001) and also compared with PE-HighPAPPA (5.9 ± 3.0 vs. 14.3 ± 4.9, p = 0.01). This pattern remained at 23–25 weeks (Figure 1). At 34–35 weeks, sFlt-1 levels increased in all four groups, increased to the greatest degree in PE-LowPAPPA women (Figure 1). At this time point, there were significant differences in sFlt-1 between NlPreg-LowPAPPA and NlPreg-HighPAPPA (10.3 ± 8.2 vs. 13.2 ± 10.7, p = 0.02) and NlPreg-LowPAPPA and PE-LowPAPPA (10.3 ± 8.2 vs. 17.2 ± 9.6, p = 0.04), Figure 1. Women with PE-LowPAPPA had the greatest change in sFlt-1 levels between the 1^st^ and 3^rd^ trimesters, compared with NLPreg-LowPAPPA and NLPreg-HighPAPPA (p < 0.02). Although PE-High PAPPA women had the highest 3^rd^ trimester sFlt-1 levels, due to the small sample size, this level was not significantly different than the other groups.

**Figure 2:**
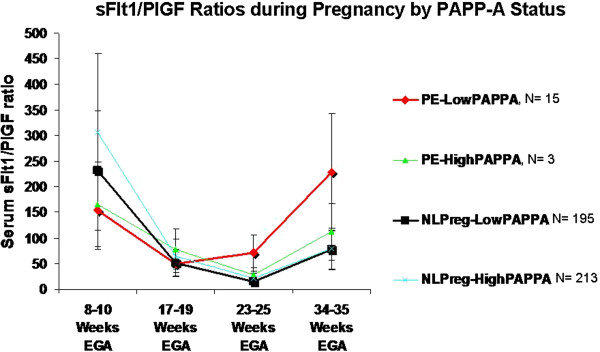
**Longitudinal sFlt-1/PlGF ratios during pregnancy by PAPP-A status and pregnancy diagnosis.** sFlt-1/PlGF ratios varied significantly between the 4 groups and overall followed a U-shaped pattern, with the greatest differences observed at 8–10 weeks and 34–35 weeks. At 8–10 weeks, PE-LowPAPPA women had significantly lower sFlt-1/PlGF ratio compared with NlPreg-LowPAPPA (p = 0.03) and also compared with NlPreg-HighPAPPA (p = 0.0006). NlPreg-LowPAPPA women also had significantly lower sFlt-1/PlGF ratio compared with NlPreg-HighPAPPA women (p < 0.0001). By 34–35 weeks, however, PE-LowPAPPA women had the highest sFlt-1/PlGF ratio, and this was significantly higher than NlPreg-LowPAPPA (p = 0.03) and NlPreg-HighPAPPA (p = 0.04). At this time point, no differences were observed in sFlt-1/PlGF ratio between NlPreg-LowPAPPA and NlPreg-HighPAPPA women.

PlGF levels did not differ significantly between these four groups (PE-LowPAPPA, PE-highPAPPA, NlPreg-LowPAPPA, Nlpreg-HighPAPPA) at each of the four time points (data not shown). However, sFlt-1/PlGF ratios did vary significantly between the 4 groups and overall followed a U-shaped pattern, with the greatest differences observed at 8–10 weeks and 34–35 weeks (Figure 2). At 8–10 weeks, PE-LowPAPPA women had significantly lower sFlt-1/PlGF ratio compared with NlPreg-LowPAPPA (154 ± 93 vs. 232 ± 143, p = 0.03) and also compared with NlPreg-HighPAPPA (154 ± 93 vs. 307 ± 206, p = 0.0006), Figure 2. NlPreg-LowPAPPA women also had significantly lower sFlt-1/PlGF ratio compared with NlPreg-HighPAPPA women (232 ± 143 vs. 307 ± 206, p < 0.0001). By 34–35 weeks, however, PE-LowPAPPA women had the highest sFlt-1/PlGF ratio, and this was significantly higher than NlPreg-LowPAPPA (228 ± 331 vs. 77 ± 220, p = 0.03) and NlPreg-HighPAPPA (228 ± 331 vs. 79 ± 211, p = 0.04). At this time point, no differences were observed in sFlt-1/PlGF ratio between NlPreg-LowPAPPA and NlPreg-HighPAPPA women.

### Demographic characteristics of women with low first trimester PAPP-A levels

There were no differences in age or parity by dichotomized PAPP-A status. With respect to 1^st^ trimester BMI, women who developed preeclampsia had significantly higher BMI, but this did not differ by PAPP-A status (PE-LowPAPPA 29.5 ± 7.0 vs. PE-highPAPPA 29.4 ± 2.9 kg/m^2^ and NlPreg-LowPAPPA 25.4 ± 5.3 vs. NlPreg-HighPAPPA 25.0 ± 5.0 kg/m^2^). Similarly, women with preeclampsia had lower gestational age at delivery compared with women with normal pregnancy, but this did not differ by PAPP-A status (PE-LowPAPPA 38.4 ± 2.3 vs. PE-HighPAPPA 38.7 ± 0.6 weeks EGA and NlPreg-LowPAPPA 39.3 ± 1.2 vs. NlPreg-HighPAPPA 39.4 ± 1.2 weeks EGA). MAP throughout pregnancy differed by pregnancy outcome but not PAPP-A status, and was significantly higher in women who developed preeclampsia (data not shown).

In contrast, PAPP-A status was significantly associated infant birth weight and the z-score of the birth weight, which was adjusted for weeks EGA. The birth weights of infants born to women with normal pregnancies differed by PAPP-A status, and lower PAPP-A MOM was associated with lower birth weight (NlPreg-LowPAPPA 3.3 ± 0.4 vs. NlPreg-HighPAPPA 3.5 ± 0.4 kg, p = 0.001). Similarly, the zscore of the birth weight, which was adjusted for weeks EGA, was also lower in women with normal pregnancy with low 1^st^ PAPP-A (NlPreg-LowPAPPA 0.04 ± 0.8 vs. NlPreg-HighPAPPA 0.3 ± 0.8, p = 0.01). In contrast, women who developed preeclampsia delivered infants with significantly lower birth weight, but this did not differ according to PAPP-A status (PE-LowPAPPA 3.1 ± 0.8 vs. PE-HighPAPPA 3.0 ± 0.3 kg).

## Discussion

Women who later developed preeclampsia demonstrated significantly lower 1^st^ trimester PAPP-A and PAPP-A MOM compared with women with normal pregnancies, as has been demonstrated previously [7]. Similarly, 1^st^ trimester sFlt-1 levels were significantly lower in women with later preeclampsia, and were highest in these women prior to delivery. sFlt-1/PLGF ratio followed a similar pattern to sFlt in these women throughout pregnancy with the lowest 1^st^ trimester ratio and highest 3^rd^ trimester ratio in women with later preeclampsia. Although a positive correlation was observed between 1^st^ trimester PAPP-A MOM and serial sFlt-1 levels throughout pregnancy, this relationship only attained statistical significance in women with normal pregnancy. PAPP-A levels were independent from other angiogenic factors and appeared to be better than first trimester sFlt-1 in predicting preeclampsia and at least as good as the first trimester sFlt-1/PlGF ratio. Based on these results, it appears that measuring first trimester sFlt-1 and/or PlGF would not improve the ability to predict development of preeclampsia beyond information obtained from first trimester PAPP-A levels.

Low PAPP-A MOM status (< median PAPP-A MOM) predicted decreased odds of normal pregnancy, while high PAPP-A status significantly predicted increased odds of normal pregnancy. Despite its correlation to 1^st^ trimester PAPP-A, low 1^st^ trimester sFlt-1 did not have the same ability to predict pregnancy outcome. However, the 1^st^ trimester sFlt-1/PlGF ratio was able to predict pregnancy outcome, though the predictive value was slightly weaker than for PAPP-A MOM. Low PAPP-A MOM status in women with normal pregnancy was also associated with lower birth weight, both measured birth weight and birth weight adjusted for gestational age.

Although PAPP-A levels have been measured to indicate risk for trisomy 21, 18 and 13 [5], research suggests that low levels also indicate risk of other pregnancy complications, including preeclampsia [6,7]. Prior studies have demonstrated that the positive predictive value of PAPP-A for preeclampsia is low [6,7], however they included women with pre-gestational or gestational diabetes, which may have affected both PAPP-A levels and pregnancy outcome. In contrast, our study focused on two carefully selected populations and compared women with confirmed normal pregnancies and with women with preeclampsia, without gestational or pregestational diabetes.

During pregnancy, PAPP-A is synthesized and released by the syncytiotrophoblast and maternal serum PAPP-A levels increase throughout pregnancy [15]. PAPP-A is thought to promote growth and development of the placenta by increasing local bioavailability of insulin-like growth factors 1 and 2 (IGF-1 and IGF-2) [16,17]. Interestingly, low 1^st^ trimester PAPP-A status in our subject population predicted lower birth weight of babies born to women with normal pregnancies, which supports findings from previous studies [18].

Research suggests that excess placental production of sFlt-1, a truncated splice variant of the VEGF receptor, contributes to the development of preeclampsia. sFlt-1 binds to and antagonizes pro-angiogenic factors such as VEGF and PLGF [1]. sFlt-1 production is upregulated in placenta of women with preeclampsia, leading to elevation of maternal serum sFlt-1 [19]. Increase in maternal sFlt-1 precedes clinical signs of preeclampsia, and maternal sFlt-1 levels correlate with disease severity [8]. However, published studies indicate an inconsistent relationship between maternal 1^st^ trimester sFlt-1 levels and development of preeclampsia, with some studies showing that 1^st^ trimester levels are increased in preeclampsia [20-22] and other studies demonstrating no differences in 1^st^ trimester sFlt-1 levels between patients who developed preeclampsia and those who had normal pregnancy [23,24]. To our knowledge, only two other published studies has presented sFlt-1 data as early as 8 weeks in pregnancy, and these studies also reported significantly lower sFlt-1 levels in pregnancies that later developed preeclampsia [4,25].

One other publication explored the relationship of 1^st^ trimester PAPP-A with 1^st^ trimester sFlt-1 in preeclampsia and normal pregnancies [26]. In that population, PAPP-A MOM levels were lower in women who later developed preeclampsia, similar to our own findings, but sFlt-1 MOM levels were higher in women who developed preeclampsia. That study examined women later in pregnancy than our study (12 weeks EGA vs. 8–10 weeks EGA in our study), and did not measure sFlt-1 levels in the 2^nd^ and 3^rd^ trimesters, when the rise in sFlt-1 has been shown to be significantly associated with the development of preeclampsia. In addition, in this paper, we report measured sFlt-1 levels, rather than sFlt-1 MOM levels, as our subjects completed laboratory measurements at specific, narrow time points. Furthermore, while it is accepted practice to measure PAPP-A in terms of MOM adjusted for gestational age and maternal weight [5], the appropriateness for this type of adjustment of sFlt-1 is less clear with most published studies reporting measured sFlt-1 levels, rather than MOM.

Our study did not find a significant relationship between 1^st^ trimester PAPP-A levels and serial PlGF levels throughout pregnancy. Other studies have examined the ability of PAPP-A and PlGF in combined models to predict preeclampsia, with some reporting a relationship between these two factors [27] and other not reporting a relationship [26]. As both PAPP-A and PlGF are thought to be involved in placental growth and development, one might expect that their levels would correlate. Based on our data, however, it appears that PAPP-A levels follow a similar pattern in early pregnancy to sFlt-1.

There may be multiple connections linking PAPP-A and sFlt-1 to each other and to preeclampsia. Both PAPP-A and sFlt-1 are produced by vascular smooth muscle cells [28,29] and may serve as markers of maternal or placental vascular dysfunction. Also, the placental renin-angiotensin-aldosterone system (RAAS) may be a common factor connecting PAPP-A and sFlt-1 to the pathophysiology of preeclampsia. During pregnancy, circulating PAPP-A is largely bound to the proform of Major Basic Protein (ProMBP) [30], which also forms a covalent complex with angiotensinogen [31]. This form of angiotensinogen is the predominant form identified in patients with preeclampsia [32]. It has been speculated that the formation of the ProMBP-PAPPA complex and formation of ProMBP-angiotensinogen complex may be competing reactions [32,33]. Research indicates that sFlt-1 is produced with RAAS activation, as pregnant mouse models have shown that infused Angiotensin II (Ang II) regulates sFlt-1 production via activation of the Ang II receptor Type 1 (AT1 receptor) [34]. As a result, it seems similar patterns in PAPP-A and sFlt-1 levels in pregnancy may reflect their common connections to maternal or placental vascular dysfunction or the placental RAAS.

This paper has several strengths. All subjects were studied at multiple, pre-specified time points throughout all three trimesters of pregnancy. The diagnosis of preeclampsia was confirmed in each subject by expert panel review of the medical record. Furthermore, all subjects were healthy prior to pregnancy, with no other significant medical diagnoses, such as gestational diabetes, that may have predisposed them to pregnancy complications. In addition, serial angiogenic markers were obtained in each subject at all time points to establish a pattern of angiogenic markers throughout pregnancy.

There were some limitations for this study as well. The number of cases of preeclampsia was relatively low, but represented 4% of the total study sample in this analysis, which is consistent with other studies and the reported prevalence of preeclampsia [1]. Many of the subjects in this study delivered after 37 weeks EGA, which may represent a different severity or phenotype of preeclampsia than other published studies. In addition, as PAPP-A levels were obtained solely for the purpose of 1^st^ trimester screening, serial PAPP-A levels throughout pregnancy were not available for analysis. It would have been interesting to explore the relationship of PAPP-A levels in the 2^nd^ and 3^rd^ trimesters with sFlt-1 levels at those time points, but that data were not available.

## Conclusions

Both 1^st^ trimester PAPP-A levels and sFlt-1 levels were significantly lower in women who later developed preeclampsia. 1^st^ trimester PAPP-A levels were significantly correlated with serial sFlt-1 levels measured longitudinally throughout pregnancy. Furthermore, low PAPP-A status in the 1^st^ trimester significantly predicted decreased odds of normal pregnancy.

## Competing interests

Dr. Karumanchi is a co-inventor of multiple patents related to angiogenic proteins for the diagnosis and therapy of preeclampsia. These patents have been licensed to multiple companies. Dr. Karumanchi reports having served as a consultant to Roche and Beckman Coulter and has financial interest in Aggamin LLC. The remaining authors report no conflicts.

## Authors’ contributions

TM conceived the study. AS performed data analysis and drafted the manuscript. TM and LWH made substantial contributions to acquisition of data, and ES, JRE and SAK provided critical review of the manuscript. All authors read and approved the final manuscript.

## Sources of funding

Supported in part by NIH Grants K12HD051959-07 (ARS), K24HL096141 (EWS), and an unrestricted grant from Abbott Diagnostics (TFM). SAK is an investigator with the Howard Hughes Medical Institute.

## Pre-publication history

The pre-publication history for this paper can be accessed here:

http://www.biomedcentral.com/1471-2393/13/85/prepub
